# Dispensers and the Insurance Bill

**Published:** 1911-11-04

**Authors:** 


### The Editor's Letter Box.

### DISPENSERS AND THE INSURANCE BILL.

"An Apothecary's Assistant" writes: I have read with much interest the letter in The Hospital of October 28, and am glad to find that at last someone has written on behalf of apothecaries' assistants, who, if they do not bestir themselves, will be wiped out of existence. At the present time, as your correspondent states, the Assistants of the Society of Apothecaries are the oldest body of qualified dispensers in the country, and they are entirely ignored by the National Insurance Bill. Why is this? I do not think we have far to seek for the reason. For some years past the Pharmaceutical Society have been improving their organisation, and as a result of this work their Parliamentary Secretary, Mr. W. S. Glyn-Jones, was elected member of Parliament for Stepney at the last General Election, and when Mr. Lloyd George introduced his National Insurance Bill into the House of Commons, steps were at once taken by the chemists of the country to look after their interests, and a series of resolutions were formulated embodying the views of the chemists of the country. Each local association discussed the matter and approached the local members of Parliament, with the result that Clauses 13 and 14 were amended in Committee on lines that were very partial to the interests of the chemists of the country. Contrast the action of the chemists with that of the certificated dispensers of the Apothecaries' Hall, and what do we see? The latter have done absolutely nothing. The Association of the members and the Secretary of the Society have been apparently asleep, and have taken no action whatever. They have an exceedingly good case, but have allowed judgment to go by default. As your correspondent has said, the assistants' qualification of the Society of Apothecaries is the oldest dispensing qualification in the country, and a large number of the assistants are acting as dispensers in Poor Law infirmaries, hospitals, and asylums, and to medical men throughout the country, where they have the management and responsibility of the dispensing of prescriptions entirely *without any supervision whatever*, and the work has been done skilfully and carefully and with satisfaction to the prescriber and the patient. Surely in any alteration of the law these persons should receive consideration, and whatever alteration might be suggested for the future their vested interests should be protected. This has always been done on previous occasions, and if we go back to 1868 we find that anyone in business as a chemist was able to register as chemist, and again when the Dentists Act was passed all who had done any dental work whatever were allowed to register—and many chemists with very little experience of dentistry proper did so register. One might further quote the Midwives Acts and Medical Act, 1858, etc. It is true that Clause 14 (5) (iii) provides that dispensers of three years' standing may be employed under the supervision of a pharmacist, but it is an insult to suggest that a certificated dispenser, who has been all his life employed in dispensing medicines, should be supervised by a chemist and druggist who has practically done no dispensing, for it is notorious that the majority of the chemists do anything but dispense; and the clause was simply inserted because it was known that the chemists of the country would be unable to do the work without such help. This being so, it is only right and just the certificated dispensers of the Apothecaries' Hall should receive due recognition, and I would suggest that Clause 14 (5) (iii), should read as follows (the alterations being printed in italics) :

" Subject to the foregoing provision as to dispensing by a medical practitioner, the regulations shall prohibit arrangements for the supply of drugs and medicines being made with persons other than persons, firms, or bodies corporate entitled to carry on the business of a chemist and druggist under the provisions of the Poisons and Pharmacy Act, 1908, *or by a certified assistant of the Society of Apothecaries,* 1815, *who for seven years immediately prior to the passing of this Act has acted as a dispenser to a duly qualified medical practitioner,* who undertake that all medicines supplied by them to insured persons shall be dispensed either by a registered pharmacist, *or by a certified assistant of the Society of Apothecaries,* 1815, or by a person who for three years immediately prior to the passing of this Act has acted as a dispenser to a duly qualified medical practitioner or a public institution."

If the clause were amended on the lines I suggest the existing dispensers would be fully safeguarded and the safety of the public protected strictly in accordance with precedent; otherwise considerable hardship will be entailed on a large number of dispensers to medical men; whereas if the clause were amended as indicated dispensers of fifty years of age, who will only be able to find situations at very small salaries, would have the opportunity of supplying medicines as they have been doing all their lives past, and thus be able to earn a livelihood.

Your correspondent omitted to state that the Poison and Pharmacy Act, 1908, provided that the Pharmaceutical Society could make by-laws for the registration without examination of persons holding Colonial diplomas or certified assistants to apothecaries under the Apothecaries Act, 1815; but so far no by-laws have been made, and surely if ever the Pharmaceutical Society intends making them, now is the opportunity. Again, the Pharmacy Act of the Isle of Man, 1900, provided that chemists' assistants who had been employed for nine years as such should be allowed to register as chemists and druggists, provided they passed the assistants' examination of the Society of Apothecaries. These facts prove conclusively that apothecaries' assistants are competent to carry on the dispensing of medicine, and should be allowed under the National Insurance Bill to follow their calling, which will otherwise be taken away from them.

In conclusion, I would suggest, that as the Association of Apothecaries' Assistants is taking no action, that every apothecary's assistant throughout the length and breadth of the land should write to the local member of Parliament and point out how adversely the National Insurance Bill will affect him, and ask the member's support to the amendment of Clause 14 (5) (iii) on the lines indicated Should we be unsuccessful in our efforts, I would suggest that the Privy Council be asked to bring pressure to bear on the Council of the Pharmaceutical Society to form by-laws for the recognition of apothecaries' assistants in accordance with the Poison and Pharmacy Act, 1908.

